# Association between non-alcoholic fatty pancreatic disease (nafpd) and the metabolic syndrome: case–control retrospective study

**DOI:** 10.1186/1475-2840-12-77

**Published:** 2013-05-20

**Authors:** Wan-Chen Wu, Chih-Yuan Wang

**Affiliations:** 1 Division of Endocrinology, Department of Internal Medicine, National Taiwan University, 7, Chung-Shang South Road, Taipei, Taiwan; 2 Health Management Center, Department of Internal Medicine, National Taiwan University Hospital, College of Medicine National Taiwan University, 7, Chung-Shang South Road, Taipei, Taiwan

**Keywords:** Non-alcoholic fatty pancreatic disease, Metabolic syndrome, Trans-abdominal ultrasonography, Obesity, Diabetes mellitus

## Abstract

**Background:**

Fatty liver is associated with insulin resistance, dyslipidemia, and obesity and is therefore considered a phenotype of metabolic syndrome. However, less is known regarding the metabolic abnormalities associated with non-alcoholic fatty pancreatic disease (NAFPD; fatty pancreas). The present study was performed to ascertain whether fatty pancreas is associated with specific metabolic risk factors and with metabolic syndrome as defined by the Adult Treatment Panel III.

**Methods:**

Five-hundred-fifty-seven healthy and consecutive subjects without known hypertension or diabetes and who received a health investigation at the National Taiwan University Hospital Health Management Center were enrolled in this retrospective study. Fatty pancreas was diagnosed via trans-abdominal ultrasonographic findings.

**Results:**

Seventy-two (12.9%) subjects diagnosed with fatty pancreas comprised the fatty pancreas group, and remaining subjects comprised the normal pancreas group. The presence of various demographic and metabolic risk factors was recorded for all subjects, and the two groups were examined for statistically significant differences in these factors. As compared to the absence of fatty pancreas**,** the presence of the disease was associated with older age and with higher values for each of the following: BMI, abdominal girth/height, abdominal girth (both genders), fasting and postprandial blood glucose, HbA1c, total cholesterol, triglycerides, LDL-cholesterol, systolic blood pressure, and platelet count. In contrast to previously reported findings, serum amylase values were lower in the fatty pancreas as compared to the control group.

**Conclusion:**

The presence of fatty pancreas represents a meaningful manifestation of metabolic syndrome together with obesity.

## Introduction

**Figure 1:**
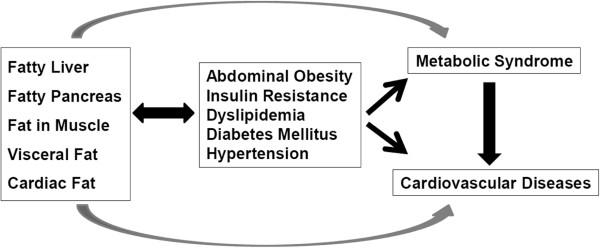
The association between ectopic fat deposition and metabolic syndrome.

Unlike fat stored in subcutaneous adipocytes, visceral fat and ectopic fat stored in tissues such as liver, heart, muscle, and pancreas are both associated with obesity and/or insulin resistance [1,2]. Fatty liver is reported to be associated with insulins resistance, dyslipidemia, and obesity and is therefore considered a phenotype of metabolic syndrome [3-5]. However, only a few studies of the metabolic abnormalities associated with non-alcoholic fatty pancreatic disease, termed NAFPD or fatty pancreas, have been performed, and the clinical consequences of fatty pancreas remain unclear (Figure 1).

Ogilvie, who first described fatty infiltration of the pancreas in 1993, reported 17% pancreatic fat storage for obese cadavers as compared with 9% for lean ones [6]. Findings from studies with animals reveal that pancreatic steatosis promotes islet cell abnormalities resulting in hyperglycemia [7-9]. Additionally, findings from older autopsy and transabdominal ultrasonography studies reveal an association of fatty pancreas with advanced age, obesity, and increased body fat [10]. Results of more recent studies indicate additional possible associations for fatty pancreas including male gender, age older than 60 years, hypertension, hepatic steatosis, alcohol use, increased body mass index (BMI), impaired insulin secretion, insulin resistance, visceral fat deposition, elevated triglycerides (TG), increased alanine aminotransferase (ALT) values, β-cell dysfunction, and diabetes [11-17]. Lee *et al.*[10,13] and Sepe *et al.*[10,13] suggested that fatty pancreas is associated with an increased frequency of metabolic syndrome and correlates with the number of parameters of metabolic syndrome. The present study was undertaken to ascertain whether NAFPD is associated with specific demographic and metabolic risk factors and with metabolic syndrome as defined by the National Cholesterol Education Program Adult Treatment Panel III (ATP III).

## Materials and methods

### Patients

Five-hundred-fifty-seven healthy and consecutive subjects not known to have hypertension or diabetes and who had a health investigation at the National Taiwan University Hospital Health Management Center were analyzed in this retrospective study during January and March, 2010. The retrospective and cross-sectional analysis study was approved by the Institutional Review Board of National Taiwan University Hospital, and this study was carried out without any linkage of personal information for all participants. All subjects received abdominal ultrasonography by gastro-hepatology specialists blinded to all information concerning these subjects during health investigation. All patients completed ultrasonographic investigation by a convex 3.5-MHz transducer (HPM2410A, Hewlett Packard, Andover, MA, USA). Usually, non-alcoholic fatty liver disease (NAFLD) diagnostic criteria included characteristic echo patterns of hepatorenal contrast, bright liver, deep attenuation, and vascular blurring. Fatty pancreas was diagnosed when there was an increase in echogenicity of the pancreatic body over that of the kidney. As the pancreas could not be compared simultaneously with the kidney in the same sonographic window, the investigators compared the difference between hepatic and renal echogenicity, and between hepatic and pancreatic echogenicity to obtain an objective pancreato-renal echo contrast [10]. Subjects were divided into the fatty pancreas group (*n* = 72) and the normal pancreas group (*n* = 485) according to the ultrasonographic diagnosis.

### Physical and biochemical findings

The height, weight, abdominal girth (AG), pulse rate, systolic blood pressure (SBP), and diastolic blood pressure (DBP) of all subjects were measured routinely in the health check-up program. AG was measured in the midsection between the lowest rib and the iliac crest of the pelvis and horizontal to the ground with the subject in an upright position. BMI and AG/height were determined for all subjects. Blood samples were tested routinely after an 8 h fast for complete blood cell count (CBC), liver function, amylase concentration, fasting blood glucose (FBG) concentration, HbA1c, total cholesterol, triglyceride (TG) concentration, high density lipoprotein cholesterol (HDL-C), and low density lipoprotein cholesterol (LDL-C). Two h postprandial blood glucose (PBG) measurements were also performed routinely.

### Definition of metabolic syndrome

The criteria for diagnosis of metabolic syndrome were those of the National Cholesterol Education Program Adult Treatment Panel III (ATP III). Metabolic syndrome was diagnosed when three or more of the following were present: abdominal obesity (waist circumference ≥ 90 cm for males or ≥ 80 cm for females), increased TG concentration (≥150 mg/dL), decreased HDL-C (< 40 mg/dL for males or < 50 mg/dL for females), hypertension (systolic blood pressure, SBP ≥ 130/diastolic blood pressure, DBP ≥ 85 mm Hg), and impaired FBG concentration (≥110 mg/dL).

### Statistical analyses

Descriptive data are presented as means ± SD for continuous variables. Differences between the fatty pancreas and normal pancreas group were determined using the Student’s *t* test. The χ^2^ test was used to evaluate the relationship between fatty pancreas and metabolic syndrome. A *p* value < 0.05 was considered statistically significant. All statistical analyses were performed with SPSS 12.0.

## Results

A total of 557 individuals, 315 men and 242 women, without known hypertension or diabetes were included. Among them, 72 were diagnosed with fatty pancreas according to the trans-abdominal ultrasonographic findings; 30 (41.7%) were male and 42 (58.3%) were female. These subjects constituted the fatty pancreas group. The remaining 485 subjects, of whom 285 were male and 200 were female, comprised the normal pancreas group.

Table 1 presents the demographic characteristics and laboratory findings for the two groups. As compared to the normal pancreas group, the fatty pancreas group was characterized by a significantly higher mean age, BMI, and AG in both males and females, by significantly higher FBG, PBG, HbA1c, total cholesterol, TG, and LDL-C values, and by a significantly higher platelet count (all *p* <0.03). By contrast, no differences in HDL-C or DBP between the two groups were observed. Interestingly, serum amylase values were lower in the fatty pancreas group as compared to the normal pancreas group (*p* <0.01).

**Table 1:** Demographic characteristics and laboratory findings for the study population

	**Total (n=557)**	**Fatty pancreas (n=72)**	**Normal pancreas (n=485)**
Gender (M:F)	315:342	30:42	285:200
Age (years^)*^	50.8±12.4	57.3±12.4	20.7±12.8
FBG (mg/dl)		104.7±29.4	95.1±20.6
AG (cm)^*^		89.8±8.1	84.3±9.4
SBP (mm HG)^#^		123.2±18.1	118.3±16.8
DBP (mm Hg)		72.7±11.2	70.1±11.1
TG (mg/dl)^*^		142.5±81.8	111.5±70.9
HDL-C (mg/dl)		46.8±10.4	48.8±11.5
Amylase(U/L)^*^		93.2±26.6	102.3±36.2
HbA1c (%)^*^		5.8±0.9	5.5±0.7
BMI^*^		25.3±2.7	23.7±3.5
Girth/Height^*^		0.56±0.05	0.50±0.06
PLT (×10)^*^		263.7±64.7	242.3±56.9

^*^p<0.01, #: p=0.023.

FBG, fasting blood glucose concentration; PBG, postprandial blood glucose concentration; AG, abdominal girth; SBP, systolic blood pressure; DBP, diastolic blood pressure; TG, triglycerides; HDL-C, high density lipoprotein-cholesterol; PLT, platelets.

No statistically significant differences between the two groups were observed for liver function tests involving aspartate aminotransferase (25.6 ± 10.3 U/L *vs.* 25.5 ± 9.3 U/L, *p* = 0.92), alanine aminotransferase (31.4 ± 27.9 U/L *vs.* 29.1 ± 19.4 U/L, *p* = 0.39), and γ-glutamyl transpeptidase (22.0 ± 16.7 U/L *vs.* 24.5 ± 28.0 U/L, *p* = 0.46) measurements or for tumor markers including carcinoembryonic antigen (0.9 ± 0.6 ng/mL *vs.* 1.1 ± 0.7 ng/mL, *p* = 0.06) and carbohydrate antigen 19–9 (12.5 ± 14.9 U/mL *vs.* 13.0 ± 0.7 U/mL, *p* = 0.65).

The prevalence of each parameter of metabolic syndrome among subjects in each group is presented in Table 2. Greater abdominal obesity, increased incidence of hypertension, higher TG values, decreased HDL-C values, and higher FBG concentrations were found for subjects with, as compared to without, fatty pancreas (all *p* <0.01).

**Table 2:** Prevalence of each parameter of metabolic syndrome in the study group

**Metabolic parameters**	**Fatty pancreas n (%)**	**Normal pancreas n (%)**
Abdominal obesity^*^	52 (72.2%)	214 (44.1%)
TG^*^	28 (38.9%)	93 (19.2%)
HDL^*^	32 (44.4%)	161 (33.2%)
HTN^*^	24 (33.3%)	128 (26.4%)
FBG^*^	34 (47.2%)	95 (19.6%)

^*^p <0.01.

Abbreviations: TG, triglycerides; HDL, high density lipoproteins; HTN, hypertension; FBG, fasting blood glucose.

The five criteria of the metabolic syndrome as established by the National Cholesterol Education Program’s Adult Treatment Panel III are: abdominal obesity (waist circumference ≥ 90 cm for males or ≥ 80 cm for females), hypertension (SBP ≥ 130/DBP ≥ 85 mm Hg), triglyceride concentrations ≥150 mg/dL, HDL-cholesterol <40 mg/dL for males or <50 mg/dL for females, and fasting glucose concentrations ≥110 mg/dL.

Each of the two study groups was examined for the percentages of subjects displaying varying degrees of parameters for metabolic syndrome (Table 3). The percentages of subjects displaying 1, 2, 3, 4 or 5 parameters and the mean number of parameters were both higher for the fatty pancreas group (all *p* <0.01). Among the 110 subjects diagnosed with metabolic syndrome (presence of 3 or more parameters), a significantly greater percentage were also diagnosed with fatty pancreas (*p* <0.01).

**Table 3:** Number of parameters of metabolic syndrome for subjects in the two groups

**Number of metabolic**	**Fatty pancreas**	**Normal pancreas**
**Syndrome parameters**	**n (%)**	**n (%)**
0	4 (5.6%)	156 (32.2%)
1	22 (30.6%)	132 (27.2%)
2	21 (29.2%)	112 (23.1%)
3	16 (22.2%)	54 (11.1%)
4	7 (9.7%)	27 (5.6%)
5	2 (2.8%)	4 (0.8%)
Meet ≥ 3 criteria^*^	25 (34.7%)	85 (17.5%)
Mean number^*^	2.1 ± 1.2	1.3 ± 1.2

*p <0.01.

The five criteria of the metabolic syndrome as established by the National Cholesterol Education Program’s Adult Treatment Panel III are: abdominal obesity, hypertension, triglyceride concentrations ≥150 mg/dL, HDL-cholesterol <40 mg/dL for males or <50 mg/dL for females, and fasting glucose concentrations ≥110 mg/dL.

## Discussion

The present study reveals significant associations of fatty pancreas with aging, obesity, systolic hypertension, hyperglycemia, and dyslipidemia. As compared to subjects without fatty pancreas, a significantly greater percentage of subjects with this condition were also diagnosed with metabolic syndrome. It should be noted that all subjects in this study received abdominal ultrasonography by gastrointestinal specialists blinded to all demographic and laboratory findings for these subjects.

Controversy has surrounded the association of fatty pancreas with advanced age. For example, findings from endoscopic ultrasonography (EUS) [17] and autopsy [18] studies are consistent with this association. However, no such association was observed in another prospective EUS study [10].

### Association between NAFPD and the parameter of metabolic syndrome

The definition criteria for metabolic syndrome are thought not ideal, and controversies continue over the validity of its predictive value for cardiovascular events and diabetes due to several factors as no inclusion of age, gender, LDL-C, family history or past history of cardiovascular disease and diabetes in the definition criteria. However, Tenenbaum *et al.* showed that current available evidences strongly supported that metabolic syndrome is an important clustering of cardiovascular risk factors and diabetes [19].

In the present study, fatty pancreas was found to be strongly associated with specific parameters of metabolic syndrome. For example, an association with obesity as manifested by increased BMI and AG was observed. This finding is compatible with those of an autopsy report [18] and with those of several human fatty pancreas studies involving the use of ultrasonography [13], EUS [10,11,17], CT [20], and MRI [12,14,15]. In animal study, Mathur *et al.* also documented that obese mice have heavier pancreas and more pancreatic fat, especially triglycerides, and concluded that obesity leads to fat infiltration of the pancreas [21]. In the present study, fatty pancreas was also found to be associated with systolic, but not diastolic, hypertension; this finding is in agreement with that of Choi *et al.*[17]. Additionally, fatty pancreas was found to be associated with increased FBG, PBG, and HbA1c values. In this regard it should be noted that findings of previous studies of the association between fatty pancreas and hyperglycemia are inconsistent. No association between fatty pancreas and DM and/or FBG was found in two studies [10,13] whereas in others fatty pancreas was found to correlate inversely with insulin secretion and β–cell function and directly with impaired glycemia and DM [12,14,16]. In the present study, fatty pancreas was also found to be strongly associated with hyperlipidemia as manifested by increased total cholesterol, TG, and LDL-C values; however, no association of HDL-C with fatty pancreas was observed. These findings differ somewhat from those of Lee *et al.*[13] who reported that fatty pancreas is associated with increased total cholesterol, TG, HDL, and free fatty acid concentrations but not with increased LDL-C concentrations. Sepe *et al.*[10] reported that fatty pancreas was associated with hyperlipidemia but lipid profiles were not provided in this report. In the present study, fatty pancreas was found to be strongly associated with the frequency of metabolic syndrome and to correlate with the number of the parameters of metabolic syndrome. These findings are consistent with those of previous studies [10,13].

Although central obesity had been defined as essential criteria of metabolic syndrome, Lee, *et al.* reported that the incidence of diabetes in subjects without central obesity was similar to that in subjects with central obesity in a Chinese hypertensive family cohort study. This indicated central obesity is not essential component of metabolic syndrome for predict diabetes [22]. In present study, fatty pancreas was found to be associated with increased FBG, PBG, and HbA1c values, and its predictive value of diabetes need to be further evaluated.

### Association between NAFPD and NAFLD

The fatty pancreas and normal pancreas groups did not differ with respect to liver function. No differences between the two groups were observed regarding albumin and globulin concentrations and AST, ALT, and γGT values. This finding is somewhat surprising in that fatty liver has been reported to be associated with fatty pancreas, and elevation of liver enzymes, especially of ALT, is often present in subjects with fatty liver. Findings of EUS studies showed an association of fatty pancreas with hepatic steatosis [10,11,17], and findings of one MRI study revealed a correlation of pancreatic fat content with liver fat content [15]. Furthermore, findings of a trans-abdominal ultrasonographic study showed a correlation between fatty pancreas and liver enzymes including AST, ALT, and γGT [13]. The findings of one autopsy study demonstrated that total pancreatic fat is significantly correlated with NAFLD, and fatty liver and fatty pancreas are related more significant in women [23]. In contrast, no association between pancreatic fat and liver fat was found in other MRI studies [12,16]. The ultrasonographic study also showed that about 68% of cases with fatty pancreas concurrently had fatty liver, but most subjects (97%) with fatty liver had fatty pancreas. The positive predictive value of fatty liver in fatty pancreas was around 70%, but the negative predictive value of fatty liver in normal pancreas was high to 96%. These findings suggested that fatty pancreas could be an initial indicator of ectopic fat deposition and an earlier manifestation of metabolic syndrome than fatty liver [13].

No statistically differences in markers of pancreatic carcinoma, including CEA and CA 19–9, were observed between the fatty pancreas and control groups. These findings are consistent with those of a previous study [10] in which fatty pancreas was not found to be associated with pancreatic carcinoma.

### Association between NAPFD and pancreatic enzymes

Two additional findings of the present report are of particular interest. The first is that serum amylase values were significantly lower for the fatty pancreas as compared to normal pancreas group. This finding contrasts with that of Sepe *et al.*[10] who found no association between fatty pancreas and serum amylase or lipase concentrations. It is known that elevated serum amylase levels often accompany acute pancreatitis and are occasionally caused by other conditions such as pancreatic tumors. However, few clinical studies investigating clinical implications of low serum amylase. Low serum amylase was thought due to diffuse pancreas destruction secondary to advanced pancreatitis [24,25]. Low serum amylase was also associated with insulin resistance in obese animal models [26,27]. Nakajima *et al.* showed that low serum amylase is associated with increased risk of metabolic abnormalities, metabolic syndrome, and diabetes, and provided a pancreatic exocrine-endocrine relationship in certain clinical condition [28]. Lower serum amylase values in subjects with fatty pancreas possibly reflected diffuse destruction of pancreas due to fat infiltration. The second is that the platelet count was significantly higher for the fatty pancreas as compared to normal pancreas group. To our knowledge, the present study is the first to examine this relationship. Further exploration of the associations of fatty pancreas with serum amylase and platelet count is planned.

### Previous studies focused on NAFPD

Table 4 summarizes prior findings regarding factors associated with fatty pancreas. Based on a number of currently conflicting findings, further studies of the associations of fatty pancreas with age, hyperglycemia, hyperlipidemia, fatty liver, and serum amylase are warranted. Some limitations of this study need to be noted. Owing to its cross-sectional case–control retrospective fashion, we only demonstrated the relation between fatty pancreas and metabolic syndrome, and the long-term effect of presence of fatty pancreas to metabolic syndrome was uncertain due to lack of longitudinal data. There was no fasting insulin measurement in the routine health investigation, and insulin resistance was unable to calculate.

**Table 4:** Summary of prior findings regarding factors associated with non-alcoholic fatty pancreas

**Study**	**Sample size (FP:NP)**	**Method of detection**	**Associated factors**	**No correlation**
Al-Haddad M, *et al.*[11]	120 (60/60)	EUS	BMI, hepatic steatosis, alcohol	
Sepe PS, *et al.*[10]	230 (64/166)	EUS	BMI, fatty liver, hyperlipidemia, MetS	DM, pancreatits, pancreatic cancer, age, alcohol, tobacco amylase
Choi CW, *et al.*[17]*.*	284 (174/110)	EUS	Hepatic steatosis, HTN, Alcohol, tobacco male, age>60	
Lee JS, *et al.*[13]	293 (180/113)	Trans-abd ominal US	IR, VAT, ALT, MetS	FBG, LDL-C
Heni M *et al.*[12]	51	MRI	BMI, VAT, waist circumference	Fatty liver
Lingvay I, et al. [14]	79	MRI	BMI, hyperglycemia	
Sijens PE, *et al.*[15]	36	MRI	BMI, liver fat	
Tushuizen ME, *et al.*[16]	36 (12/24)	MRI	Beta-cell dysfunction	Fatty liver, VAT

IR, insulin resistance; DM, diabetes mellitus; MetS, metabolic syndrome; ALT, alanine aminotransferase; HTN, hypertension; VAT, visceral adipose tissue; FP, fatty pancreas; NP, normal pancreas; EUS, endoscopic ultrasonography; US, ultrasonography; FBG, fasting blood glucose; LDL-C, low density lipoprotein-cholesterol.

## Conclusion

In conclusion, non-alcoholic fatty pancreatic disease (NAFPD or fatty pancreas) may represent a meaningful manifestation of metabolic syndrome. The associations of fatty pancreas with age, hyperglycemia, hyperlipidemia, fatty liver, serum amylase, and platelet count require further examination. The incidental abdominal ultrasonographic finding of fatty pancreas in a subject without known metabolic syndrome but abdominal obesity should alert further investigation. Further cohort study should be carried out to observe serial changes of fatty pancreas in diabetic and non-diabetic subjects.

## Consent

The retrospective and cross-sectional analysis study was approved by the Institutional Review Board of National Taiwan University Hospital, and this study was carried out without any linkage of personal information for all participants. Written informed consent was obtained from the patient for publication of this report.

## Competing interests

The authors declare that they have no competing interests.

## Authors’ contributions

WCW: Discussion, wrote manuscript. CYW: Researched data, discussion, reviewed/edited manuscript. All authors read and approved the final manuscript.
